# Quantification of perfusion defects with high resolution 2D and 3D adenosine stress perfusion 3.0 Tesla CMR

**DOI:** 10.1186/1532-429X-16-S1-P195

**Published:** 2014-01-16

**Authors:** Adam K McDiarmid, Manish Motwani, David P Ripley, Kevin Mohee, Sebastian Kozerke, John P Greenwood, Sven Plein

**Affiliations:** 1 LIGHT, University of Leeds, Leeds, West Yorkshire, UK; 2 Institute for Biomedical Engineering, University and ETH Zurich, Zurich, Switzerland

## Background

Myocardial ischaemic burden is an important marker of outcome in stable ischaemic heart disease with a 10% ischaemic burden suggested as a clinically relevant threshold for revascularisation. Standard CMR stress perfusion imaging utilises 3 short axis slices, each acquired in different phases of the cardiac cycle. Recently proposed 3D myocardial perfusion CMR allows the entire myocardium to be sampled within the same cardiac phase and therefore should allow more accurate quantification of ischaemic burden without extrapolation. In this study we compared visual ischaemia detection and quantification by 2D high resolution and 3D whole heart myocardial perfusion CMR.

## Methods

CMR imaging was performed on a 3T Philips Achieva TX system. Stress (Adenosine 140 mcg/kg/min) and rest acquisitions were performed (Gadovist 0.075 mmol/kg), separated by 15 min. 2D perfusion CMR used 3 slices positioned using the '3 of 5' technique (variable gap) and a spoiled gradient echo pulse sequence with 5 fold k-t SENSE acceleration, voxel size 1.06*1.06*10 mm 3. 3D perfusion used 12 contiguous slices acquired with a 3D spoiled gradient echo sequence at mid-diastole and 10 fold k-t SENSE acceleration with 11 training profiles, voxel size of 2.3*2.3*5 mm 3. 23 patients with perfusion abnormalities on 2D perfusion CMR were recalled for 3D whole heart acquisition on a separate day. Perfusion images were analysed independently by two observers. Image quality (4 = Excellent, 3 = Good, 2 = Poor, 1 = uninterpretable) and diagnostic confidence by coronary territory was graded (1 = high diagnostic confidence, 0 = low diagnostic confidence). Perfusion abnormalities were contoured in the frame of maximal remote myocardium signal intensity, and expressed as a percentage of total myocardium.

## Results

**Figure 1:**
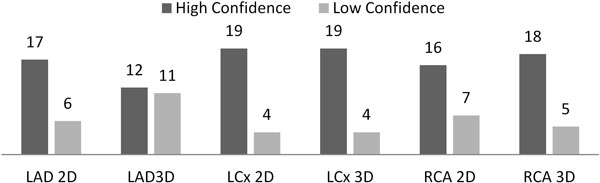
**Diagnostic Confidence by Coronary Territory**.

**Figure 2:**
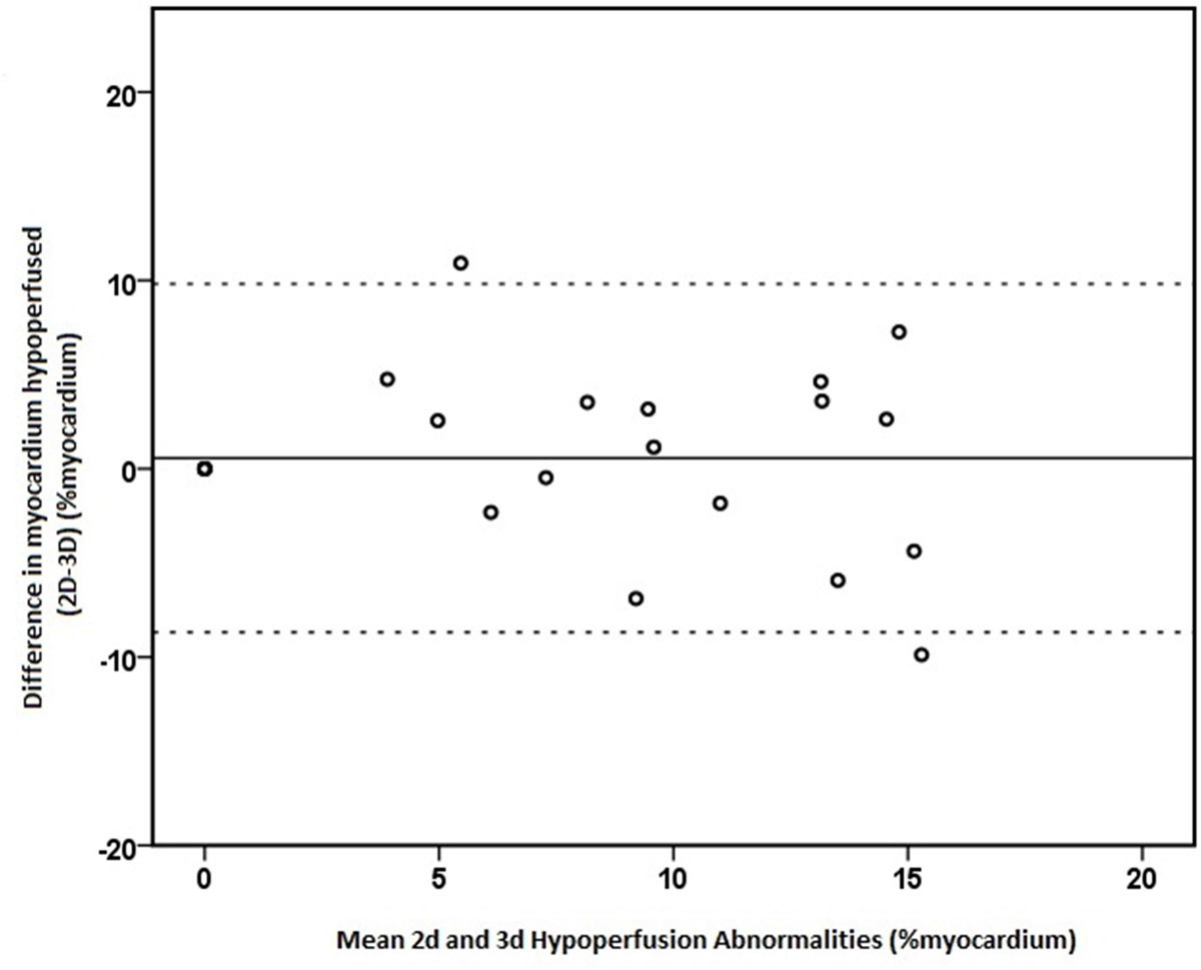
**Bland-Altman Plot, Agreement of 2D vs 3D ischaemia quantification**. Bias was 0.1% (95%CI -9.2% to 9.3%)

Of the 23 paired data sets, images in one 2D set were uninterpretable due to artefact, leaving 22 pairs for analysis. Image quality was excellent or good in 21/22(95%) for 2D and 20/22(91%) for 3D acquisitions. Diagnostic confidence expressed by coronary territory did not differ significantly in LAD, LCx or RCA [Figure 1] (p = 0.12, 1.0 and 0.74 respectively). 2D and 3D perfusion quantification agreed, with a mean bias on Bland Altman analysis of 0.7%(r2 = 0.488, p < 0.001) [Figure 2]. At the 10% threshold of ischaemic burden with either method, there was agreement in 9 of 12 sets, in non-agreement 2D quantification was greater than 3D and differed by 1.1, 3.2, and 10.9%.

## Conclusions

Image quality and diagnostic confidence are similar with both high resolution 2D and whole heart 3D myocardial perfusion CMR. No systematic bias in perfusion quantification between the two methods was detected.

## Funding

SP and JPG receive an educational research grant from Philips Healthcare. SP is funded by a British Heart Foundation fellowship.

